# Differential Associations Between Family Socioeconomic Position and Neighborhood Economic Conditions Versus Safety by Race in the United States

**DOI:** 10.65773/ssia.2.1.96

**Published:** 2025-11-26

**Authors:** Shervin Assari, Hossein Zare

**Affiliations:** 1 Charles R Drew University of Medicine and Science, Los Angeles, California, USA; 2 Johns Hopkins University, Baltimore, Baltimore, MD, USA

**Keywords:** socioeconomic position, neighborhood disadvantage, Area Deprivation Index, structural inequality, racial disparities, neighborhood crime exposure, contextual data, residential history, socioeconomic gradients

## Abstract

**Background:**

Family socioeconomic position (SEP) is often linked to neighborhood conditions, with higher SEP generally associated with more advantaged structural characteristics such as higher neighborhood income and lower poverty. Whether these associations extend similarly to neighborhood safety, and whether these patterns vary across racial/ethnic groups, remains an important but understudied question.

**Objective:**

To examine how family SEP associates to multiple dimensions of neighborhood conditions—including economic resources, poverty, and crime-related indicators—and to estimate whether these associations differ across racial/ethnic groups.

**Methods:**

We analyzed individual-level family SEP indicators in relation to neighborhood characteristics, including safety (violent offenses, drug-related offenses, drug sales, marijuana sales, drug possession, and driving under influence [DUI] events). Associations were estimated overall and separately by racial/ethnic background. Models adjusted for demographic covariates. Analyses focused on cross-sectional patterns and emphasized associations rather than mechanisms.

**Results:**

Higher family socioeconomic position was associated with more advantaged neighborhood characteristics overall and across both racial/ethnic groups. These associations were generally stronger for Black families than for White families when the outcomes reflected neighborhood income and poverty levels. In contrast, the associations between family SEP and neighborhood crime statistics were weaker for Black families than for White families. This pattern suggests that higher family SEP corresponded differently to improvements in neighborhood economic and safety characteristics for Black and White families.

**Conclusions:**

Family socioeconomic position is linked to more advantaged neighborhood environments, but the strength of these associations varies across racial/ethnic groups and depends on the neighborhood domain being examined. Economic neighborhood features appear more responsive to family SEP among Black families, whereas neighborhood safety indicators seem more responsive to family SEP among White families. These findings raise the possibility that crime metrics may partly reflect racialized policing that are not equally sensitive to socioeconomic improvements of Black and White communities.

## Introduction

Family socioeconomic position (SEP) plays a major role in shaping the living conditions and environments in which children and adults live. Higher SEP is generally associated with residential access to neighborhoods that have greater economic resources, lower poverty, and lower crime rate [1–3]. These neighborhood characteristics are often viewed as pathways through which socioeconomic advantages become embedded in daily life and influence health and wellbeing [3].

Although these patterns are well documented, much less is known about whether the strength of SEP associations varies across different types of neighborhood conditions, including measures tied to economic structure versus indicators linked to crime or policing. An equally important and often overlooked question is whether these associations differ across racial and ethnic groups. At least some evidence suggests that Black people with high SEP are more likely to remain in high-crime or high-disorder areas than their White counterparts with similar SEP [4–6].

Neighborhoods are not neutral spaces but reflect a long history of residential segregation, uneven investment, discriminatory housing practices, and racialized policing [7,8]. Because of these structural forces, families of similar SEP may not have equivalent access to comparable neighborhood environments. In many contexts, Black families with higher SEP still encounter barriers to securing neighborhoods with the same level of safety or opportunity as their White counterparts [9,10]. These patterns have been described as “unequal neighborhood returns” to socioeconomic resources [11].

According to minority threat theory, policing patterns may shift depending on the racial composition of a neighborhood. Classic work argues that when the dominant racial group is larger, political and institutional systems may devote more surveillance and social control toward minoritized groups, often in ways that reflect collective perceptions of threat rather than actual behavior [12,13]. In many U.S. settings, neighborhoods with a higher proportion of White residents tend to show stronger policing presence and enforcement, patterns that have been interpreted as aligning with efforts to protect dominant-group interests and maintain existing social hierarchies [14,15]. These dynamics may result in more frequent police contact and criminal justice exposure for Black residents, even when overall crime levels are similar [16,17]. Research has also shown that concentrated policing in communities of color is associated with increased surveillance, more stops, and broader institutional avoidance among Black residents, suggesting that unequal enforcement—not differential offending—may underlie observed disparities [18,19]. These patterns do not imply intentional harm but point to how demographic power structures may shape policing priorities in ways that reinforce racialized inequalities.

Structural neighborhood features such as median income and poverty levels are typically more stable reflections of long-term economic investment. Crime-related indicators, however, are more complex because they may reflect both actual community safety and the practices through which crime is recorded [20]. In the United States, not only the crime itself but also racialized differences in policing, surveillance, and enforcement shape the distribution of recorded crime [21,22]. Consequently, crime indicators may not respond to family SEP in the same way as structural neighborhood characteristics. For example, Black families—even at higher SEP levels—may reside in areas where residents are more frequently stopped, searched, or arrested, making crime statistics appear higher or less responsive to socioeconomic differences regardless of true safety conditions [23,24].

### 1.1. Objectives

The present study examines how family SEP relates to multiple dimensions of neighborhood conditions, including both structural economic indicators and crime-related measures, and tests whether these patterns differ by families’ race/ethnicity. By comparing the strength of these associations across groups, this work aims to clarify how socioeconomic position aligns with neighborhood environments, and whether certain neighborhood features show weaker or stronger SEP gradients for Black families relative to White families. Rather than implying causal pathways, the goal is to describe observed patterns that may reflect deeper structural processes linking socioeconomic resources, residential opportunities, and racialized systems of surveillance and enforcement.

## 2. Methods

### 2.1. Design and Participants

This study used data from the baseline wave of the Adolescent Brain Cognitive Development (ABCD) Study [25–29], a large, multisite cohort of children ages 9–10 recruited from 21 research sites across the United States. The study was designed to capture geographic, racial, and socioeconomic diversity through population-based recruitment strategies. All procedures were approved by the Institutional Review Boards at participating institutions, and both parental consent and child assent were obtained. The present analyses used the publicly available ABCD Release, which provides de-identified individual-level data and linked contextual variables. Only participants with complete family socioeconomic position (SEP) measures and contextual neighborhood data were included. When participants had multiple historical addresses, the most recent residential address at baseline was used, consistent with ABCD recommendations for capturing current neighborhood exposure [25–29].

### 2.2. Residential History and Contextual Data

Neighborhood variables in ABCD are not shared as raw ZIP codes or census identifiers for privacy protection. Instead, ABCD provides curated contextual data derived from geocoded residential addresses through a structured and secure linkage process. Residential addresses are processed through the ABCD data enclave, converted into area-level indicators, and then de-identified before release. The variables available to investigators therefore reflect neighborhood conditions but contain no geographic identifiers. This approach retains spatial meaning while meeting strict confidentiality standards [25–29].

The contextual data used in this study was based on the most recent address reported in the ABCD residential history file. These data include multiple indicators derived from census-based sources and administrative datasets, including socioeconomic, housing, and crime-related measures. The deprivation measures were constructed following the logic and core components of the Area Deprivation Index (ADI) developed by Kind and colleagues [30]. ADI incorporates indicators of neighborhood socioeconomic disadvantage such as income, employment, household structure, and housing quality. In the ABCD contextual dataset, analogous constructs are available, and we used the variables corresponding to median household income, poverty rates, single-parent household prevalence, employment levels, and related components. These indicators capture structural features of neighborhoods reflecting long-term investment, material resources, and social advantages or disadvantages [30].

In addition to structural neighborhood metrics, the dataset includes crime-related contextual variables derived from administrative sources. These include indicators of violent offenses, drug-related offenses, drug sales, marijuana sales, drug possession, and driving-under-the-influence incidents. As with the other contextual variables, these crime indicators are not provided at the ZIP-code level; rather, they represent area-level (census tract) crime exposures linked internally by ABCD and released as de-identified estimates. These variables may reflect neighborhood safety conditions as well as differences in surveillance, policing, and reporting practices across communities [20–24].

### 2.3. Family Socioeconomic Position

Family SEP was measured using parent-reported information on household income, parental educational attainment, financial hardship, and related socioeconomic indicators available in the ABCD parent surveys. These variables were treated as continuous or ordered measures to capture gradients of socioeconomic position rather than categorical status. SEP measures were standardized when combined across domains.

### 2.4. Race and Ethnicity

Race and ethnicity were obtained from parent reports. Analyses focused on differences in associations across major racial and ethnic categories, with particular attention to comparisons between Black and White families given longstanding disparities in neighborhood access and structural conditions. These categories reflect socially meaningful classifications rather than biological groupings and were analyzed to understand contextual inequalities.

### 2.5. Outcome Variables: Neighborhood Structure and Crime Indicators

The primary outcomes included neighborhood structural characteristics and crime-related contextual indicators. Structural neighborhood features reflected socioeconomic investment and included median household income, local poverty rates, and related ADI-based components [30]. Crime indicators included measures of violent offenses, drug-related offenses, drug sales, marijuana sales, drug possession events, and driving-under-the-influence counts [20–24]. All contextual variables represent de-identified area-level exposures created by ABCD’s secure data linkage and do not reveal geographic information.

### 2.6. Data Quality and Address Validity

The ABCD residential history file includes internal checks for address validity, including completeness of geocoding and the plausibility of reported addresses [25–29]. Only addresses verified and linked by the ABCD geocoding process were included. Because the contextual dataset uses the most recent verified residential address, this approach aligns with exposure contemporaneous to baseline data collection. All contextual variables used in this analysis were derived from addresses that passed ABCD’s QC procedures.

### 2.7. Statistical Analysis

All statistical analyses were conducted in SPSS (version 29). Descriptive statistics were used to summarize family SEP, neighborhood structural characteristics, and neighborhood crime indicators. Associations between SEP and neighborhood variables were examined using linear regression models. Separate models were estimated overall and stratified by racial and ethnic groups to assess whether the strength of SEP associations differed across groups. Interaction models (SEP × race/ethnicity) were estimated as a supplementary test of differential associations, though interpretation emphasized stratified results rather than statistical interaction terms. All models adjusted for child age, sex, and parental marital status to account for demographic differences that could influence neighborhood selection. Analyses were cross-sectional and focused on associations rather than causal pathways.

Residuals were examined to assess linearity and model fit. No transformations were applied to the primary variables to preserve interpretability. Sensitivity analyses tested whether excluding cases with partially missing SEP indicators altered the pattern of results; findings were unchanged.

## 3. Results

### 3.1. Socioeconomic Position

Socioeconomic characteristics differed notably by race/ethnicity, with Black families showing lower family income, lower parent education, higher financial difficulty, and lower income-to-needs ratios and SEP scores compared with White families (Table 1). Although age and household size were similar across groups, the gradients in education and income-related indicators suggest that Black children in this sample are, on average, growing up in families with fewer socioeconomic resources. These contrasts in family SEP provide important context for interpreting subsequent differences in neighborhood conditions and crime exposures.

### 3.2. Neighborhood ADI

Neighborhood structural indicators based on Area Deprivation Index (ADI) components showed consistent racial disparities, with Black children living in areas characterized by lower median family income, higher deprivation summary scores, and higher national ADI percentiles than White children (Table 2). Even though some indicators, such as the percentage employed in white-collar occupations, appeared similar or slightly higher in Black neighborhoods, the overall ADI profiles point to greater neighborhood disadvantage for Black families. These patterns indicate that differences in family SEP are accompanied by systematic differences in neighborhood-level socioeconomic conditions.

### 3.3. Neighborhood Crime

Neighborhood crime indicators derived from Uniform Crime Reports further revealed that Black children, on average, reside in areas with equal or higher levels of recorded total crime, drug-related violations, drug sales, marijuana sales, and drug possession compared with White children, despite Black families having, in some cases, similar or only modestly different neighborhood structural characteristics (Table 3). Although DUI incidents were slightly lower in areas where Black children lived, most crime-related indicators were higher, suggesting that the environments surrounding Black youth are characterized by greater exposure to recorded criminal activity. These contrasts raise questions about how structural factors, including policing and surveillance, may shape crime statistics across neighborhoods that differ by racial composition.

As shown by Table 4, family socioeconomic position indicators were consistently related to structural neighborhood characteristics. Across the full sample, higher SEP, higher parent education, higher income, and higher income-to-needs ratio were all associated with indicators of less neighborhood deprivation, including lower neighborhood poverty, unemployment, and crowding, and higher neighborhood income, home value, and home ownership (all |r| often ≥ 0.30). In contrast, higher financial difficulty showed the opposite pattern, correlating with more disadvantaged neighborhood profiles. These patterns were very similar for White families, with strong and monotonic correlations between family SEP indicators and ADI-related neighborhood measures. Among Black families, correlations between SEP and structural neighborhood variables were also in the expected direction and often moderately strong, indicating that higher SEP was linked to higher neighborhood income and lower poverty and unemployment. However, for crime indicators, the pattern differed by race. For White families, higher SEP was weakly and generally negatively associated with total crime, violent crime, and drug-related offenses (small negative correlations). Among Black families, higher SEP was instead weakly and positively associated with total crime, violent crime, and several drug-related indicators, suggesting that even at higher levels of family SEP, Black families tend to live in areas with equal or greater recorded crime and enforcement activity.

As shown by Table 5, higher family SEP was consistently associated with more advantaged neighborhood characteristics across multiple indicators, including lower poverty, unemployment, and crowding, as well as higher household income, and home ownership. Conversely, identifying as Black was associated with residence in more socioeconomically disadvantaged neighborhoods on most structural indicators, even after accounting for family SEP. The interaction between family SEP and race (High SEP × Black) revealed two major patterns. Although higher SEP predicted better economic neighborhood conditions for Black than White families, these associations were generally weaker for Black than White families for key markers of neighborhood crime-related outcomes.

## 4. Discussion

This study examined how family socioeconomic position (SEP) relates to multiple domains of neighborhood conditions and whether these associations differ across racial/ethnic groups. Several clear patterns emerged. First, higher family SEP was associated with more advantaged neighborhood characteristics overall, including higher neighborhood income and lower poverty levels. Second, these SEP gradients for structural neighborhood resources were larger for Black families than for White families, suggesting that increases in socioeconomic position may correspond to relatively strong improvements in the structural features of the neighborhoods in which Black families live. Third, and in contrast, the associations between SEP and a broad set of neighborhood crime indicators were noticeably weaker for Black families than for White families. Taken together, these findings indicate that family SEP does not translate uniformly into neighborhood advantages, and that the sensitivity of neighborhood characteristics to socioeconomic resources differs both by neighborhood domain and by race.

The stronger SEP gradients in structural neighborhood conditions observed among Black families align with the idea that socioeconomic resources remain meaningful for residential access. As families gain economic and educational resources, they may be better able to enter neighborhoods with stronger local investment, higher-performing schools, and more economic stability [1–3,7–11]. These patterns suggest that increases in SEP may correspond to visible improvements in some aspects of neighborhood opportunity. For Black families, these gains may even be more pronounced because the starting point—given historical and current inequalities in housing and neighborhood access—may involve steeper barriers and therefore potentially steeper improvements as SEP increases [7–11]. However, the fact that the same magnitude of improvement does not appear for crime-related neighborhood indicators points to a more complex set of structural processes [16–24].

The weaker association between SEP and crime exposure among Black families suggests that crime-related neighborhood indicators may not respond to family socioeconomic resources in the same way that structural features do. One possible interpretation is that crime indicators may partly reflect racialized differences in policing, surveillance, and enforcement, which are not equally sensitive to family SEP [12–15,18–24]. In many U.S. settings, neighborhoods with larger Black populations—regardless of socioeconomic profile—experience more intensive policing, more frequent stops, searches, and arrests, and greater enforcement of drug-related and low-level offenses [18,19,21–24]. If crime indicators partially capture law enforcement activity rather than underlying safety alone, then even higher-SEP Black families may continue to live in areas where policing practices elevate the appearance of crime. This would produce weaker SEP gradients in crime indicators not because crime is unresponsive to socioeconomic position per se, but because the recorded data may be shaped by systems that do not respond uniformly across racial groups.

A second interpretation is that Black families, even with higher SEP, face more constrained housing options due to ongoing forms of discrimination, racial steering, and the legacy of segregated housing markets [7–9,22]. If upward socioeconomic gains provide access to neighborhoods with better economic structures, but still limit access to neighborhoods with lower levels of recorded crime, then the neighborhood exposure profiles of higher-SEP Black families may diverge from those of higher-SEP White families. This possibility is consistent with longstanding evidence that Black families—across income levels—face structural limits in accessing neighborhoods with lower policing intensity or lower recorded crime [4,6,9,11,22,31]. In this sense, the weaker SEP–crime relationship observed for Black families may reflect fewer opportunities to translate socioeconomic gains into improvements in this specific neighborhood domain.

Another consideration is that structural neighborhood factors such as median income and poverty rate capture long-term investment patterns and infrastructure stability, which may be more directly tied to economic resources. Crime indicators, in contrast, are shaped by a combination of actual behaviors, environmental stressors, surveillance practices, and administrative reporting patterns [20–24]. As a result, crime metrics may be less responsive to individual family SEP and more reflective of broader structural conditions that differ across racial groups. This divergence between structural and crime-related indicators may help explain why neighborhood improvements linked to SEP appear domain-specific and racialized.

These findings also contribute to the broader literature on “unequal returns” to socioeconomic resources. They suggest that family SEP may produce improvements in some neighborhood domains for Black families—particularly those tied to structural investment—but that these gains do not necessarily extend to recorded crime exposure [4,6,9,11,31]. This pattern is not consistent with a uniform diminished-returns model; instead, it suggests a domain-specific pattern in which certain neighborhood characteristics are more tightly linked to family SEP than others. Understanding this complexity may be critical for clarifying how socioeconomic resources translate into lived experiences across racial groups and how structural forces may shape the environments available to families, even at higher levels of SEP.

In a related study, Xie et al. examined whether individual and neighborhood SEP align more closely in urban than in rural settings, using the association between obesity and self-rated health as an illustrative case. Drawing on two population-based surveys from eight counties in Pennsylvania, they assessed correlations between household income and neighborhood advantage and evaluated residual confounding by individual SEP in models adjusting only for neighborhood SEP. The correspondence between individual and neighborhood SEP was stronger in more urban counties, while the degree of confounding decreased with increasing urbanicity. These findings suggest that neighborhood-based SEP measures may insufficiently capture individual socioeconomic position, particularly in less urban and rural contexts [32].

Other studies in the United States and Canada indicate that alignment between individual- and neighborhood-level SEP measures differs across settings. Agreement was weaker in a mixed urban–rural county in Minnesota than in an urban county in Missouri, with Cohen’s κ values ranging from 0.15 to 0.22 versus 0.26 to 0.36, respectively [33,34]. Similar patterns have been reported in Canada, where a study in a largely rural patient cohort in Alberta found very low concordance between self-reported income and area-based income measures (κ = 0.07) [35]. In contrast, higher, though still modest, correlations were observed in metropolitan settings, including Vancouver (Spearman’s ρ = 0.23–0.35) [36] and Montreal (ρ = 0.31–0.39) [37]. Beyond North America, analysis of a pediatric asthma cohort in Rome, Italy showed moderate agreement between parental education and neighborhood-level SEP indicators (ρ = 0.47–0.48) [38].

### 4.1. Limitations

Several limitations should be considered when interpreting these findings. First, the study is cross-sectional, which prevents conclusions about temporal ordering, neighborhood selection processes, or how socioeconomic position (SEP) may accumulate over time to shape neighborhood exposure. Second, the neighborhood measures used here rely on administrative and spatially linked data, which may not fully capture residents’ lived experiences of community conditions. Crime indicators in particular reflect reported or recorded events, which can be influenced by policing patterns, surveillance intensity, and reporting practices that differ across jurisdictions [20–24]. As a result, these variables may capture law enforcement activity as much as, or more than, the underlying safety environment. Third, although the SEP measures are robust, they do not encompass the full spectrum of structural advantages and disadvantages that shape residential opportunities, including historical constraints, discrimination in housing and lending markets, zoning policies, and the availability of affordable housing [7–11,22]. Fourth, the analyses centered on associations rather than mechanisms, and the racial differences observed here should be interpreted as patterns consistent with—but not proof of—broader structural processes. Finally, the findings reflect the specific indicators available in the dataset and may not generalize to all communities or reflect the full complexity of neighborhoods across the United States.

This study examined how family socioeconomic position relates to two major dimensions of neighborhood conditions across racial/ethnic groups. The results indicate that higher SEP corresponds to clear improvements in structural neighborhood characteristics—including higher neighborhood income and lower poverty—with these associations appearing particularly strong for Black families. However, the relationship between SEP and neighborhood crime exposure was noticeably weaker for Black families than for White families, suggesting that crime-related indicators may respond differently to family socioeconomic resources of Black and White families. One interpretation is that crime measures partly reflect racialized practices in policing and surveillance, which may limit the extent to which improved socioeconomic standing translates into safer recorded neighborhood environments for Black families [12–15,18–24].

## 5. Conclusion

These findings highlight the importance of distinguishing between different domains of neighborhood conditions and recognizing that socioeconomic position at the family level may not yield uniform improvements across them. They also underscore the need to consider structural and racialized processes when interpreting neighborhood crime data, particularly when studying racial differences in access to safer environments. While the results do not identify causal pathways, they point to the possibility that socioeconomic resources translate into neighborhood advantages in unequal ways across racial groups, with implications for how environmental conditions may influence health, development, and opportunity. Future work using longitudinal data may clarify how these patterns unfold over time and the degree to which structural constraints shape the neighborhood environments available to families at different levels of socioeconomic position.

## Figures and Tables

**Table 1.** Socioeconomic Variables by Race/Ethnicity.

Variable	Race/Ethnicity	n	Min	Max	Mean	SD	P

Age (years)	White	8,255	8.00	11.00	9.48	0.51	NS
	Black	2,513	8.00	11.00	9.47	0.51	
	All	10,768	8.00	11.00	9.48	0.51	
Household size	White	8,187	0.00	19.00	4.73	1.46	NS
	Black	2,379	0.00	14.00	4.62	1.80	
	All	10,566	0.00	19.00	4.70	1.54	
Parent education (years)	White	8,259	1.00	21.00	17.14	2.47	< 0.001
	Black	2,507	3.00	21.00	15.40	2.58	
	All	10,766	1.00	21.00	16.74	2.60	
Family income (1–10)	White	7,765	1.00	10.00	7.86	1.96	< 0.001
	Black	2,171	1.00	10.00	5.36	2.70	
	All	9,936	1.00	10.00	7.31	2.38	
Financial difficulty (0–7)	White	8,199	0.00	7.00	0.31	0.89	< 0.001
	Black	2,474	0.00	7.00	0.99	1.49	
	All	10,673	0.00	7.00	0.47	1.10	
Income-to-needs ratio	White	7,704	0.06	9.00	1.81	0.70	< 0.001
	Black	2,083	0.10	8.00	1.33	0.86	
	All	9,787	0.06	9.00	1.71	0.77	
SEP composite score	White	7,702	−5.49	4.78	0.39	1.32	< 0.001
	Black	2,082	−5.08	4.11	−1.17	1.73	
	All	9,784	−5.49	4.78	0.06	1.55	
SEP based on a principal component analysis (z-score)	White	7,702	−3.44	3.00	0.24	0.83	< 0.001
	Black	2,082	−3.18	2.58	−0.74	1.09	
	All	9,784	−3.44	3.00	0.04	0.97	

**Note:** Adolescent Brain Cognitive Development (ABCD) Study, 2016–2018

**Table 2.** Neighborhood/ADI Indicators by Race/Ethnicity.

Variable	Race/Ethnicity	n	Min	Max	Mean	SD	

% adults < 9 years education ^a^	White	7,857	0.00	55.00	3.98	6.12	< 0.001
	Black	2,303	0.00	47.00	5.74	5.73	
	All	10,160	0.00	55.00	4.38	6.08	
% ≥ high school diploma ^a^	White	7,857	0.00	100.00	90.52	10.14	< 0.001
	Black	2,303	30.00	100.00	83.76	10.69	
	All	10,160	0.00	100.00	88.99	10.65	
% white-collar occupations ^a^	White	7,857	0.00	100.00	93.53	4.50	NS
	Black	2,303	67.00	100.00	94.61	4.05	
	All	10,160	0.00	100.00	93.78	4.43	
Neighborhood median family income ($)^a^	White	7,857	0	250001	84,162.54	34,491.14	< 0.001
	Black	2,303	8,546	210,455	53,259.11	28,370.71	
	All	10,160	0	250001	77,157.56	35,633.76	
ADI summary score (Kind et al.) ^a^	White	7,857	0.00	125.00	89.78	24.45	< 0.001
	Black	2,303	0.00	126.00	104.61	22.00	
	All	10,160	0.00	126.00	93.14	24.71	
ADI national percentile (0–100) ^a^	White	7,857	0.00	100.00	33.42	23.05	< 0.001
	Black	2,303	0.00	100.00	61.54	29.82	
	All	10,160	0.00	100.00	39.80	27.40	

**Note:** Adolescent Brain Cognitive Development (ABCD) Study, 2016–2018;

a Census Track, an Area Deprivation Index (ADI) element; NS; Non-Significant; p values: two tailed.

**Table 3.** Neighborhood Crime Indicators (Uniform Crime Reports) by Race/Ethnicity.

Variable	Race/Ethnicity	n	Min	Max	Mean	SD	

Total crime ^a^	White	7,857	0	348049	46,369.51	80,449.31	< 0.001
	Black	2,303	0	348049	48,399.76	67,702.39	
	All	10,160	0	348049	46,829.72	77,744.80	
Adult drug abuse violations ^a^	White	7,857	0	50,189	6,195.87	11,852.26	< 0.001
	Black	2,303	0	50,189	7,110.72	10,329.48	
	All	10,160	0	50,189	6,403.25	11,530.61	
Drug sale total ^a^	White	7,857	0	9,938	1,103.59	2,307.48	< 0.001
	Black	2,303	0	9,938	1,332.71	2,055.81	
	All	10,160	0	9,938	1,155.52	2,254.84	
Marijuana sale ^a^	White	7,857	0	3,663	408.06	849.65	< 0.001
	Black	2,303	0	3,663	405.37	709.77	
	All	10,160	0	3,663	407.45	820.00	
Drug possession ^a^	White	7,857	0	40,257	5,068.69	9,582.99	< 0.001
	Black	2,303	0	40,257	5,745.71	8,339.57	
	All	10,160	0	40,257	5,222.15	9,319.62	
DUI incidents ^a^	White	7,857	0	42,953	5,087.33	10,117.56	< 0.001
	Black	2,303	0	42,953	4,545.00	8,474.43	
	All	10,160	0	42,953	4,964.40	9,771.60	

**Note:** Adolescent Brain Cognitive Development (ABCD) Study, 2016–2018;

a Census Track, a Universal Crime Report (UCR) element

**Table 4.** Correlations Between Family SEP Indicators and Neighborhood Variables, Overall, and by Race/Ethnicity.

Variable	Z-score SEP	Parent education (yrs)	Family income (1–10)	Financial difficulty (0–7)	Income-to-needs ratio

**All**					
% adults < 9 years education a	−0.36**	−0.37**	−0.36**	0.14**	−0.23**
% ≥ high school diploma a	0.48**	0.46**	0.48**	−0.21**	0.32**
% white-collar occupations a	0.10**	0.13**	0.07**	−0.02*	0.12**
share of households ≥ $50k a	0.44**	0.37**	0.45**	−0.23**	0.30**
median family income a	0.52**	0.45**	0.54**	−0.26**	0.36**
income disparity (Singh) a	−0.47**	−0.38**	−0.51**	0.25**	−0.29**
median home value a	0.38**	0.30**	0.39**	−0.21**	0.31**
median gross rent a	0.27**	0.21**	0.28**	−0.16**	0.22**
median monthly mortgage a	0.40**	0.32**	0.42**	−0.22**	0.33**
% owner-occupied a	0.36**	0.32**	0.40**	−0.19**	0.19**
% crowding (>1 person/room) a	−0.27**	−0.30**	−0.27**	0.10**	−0.18**
% unemployed a	−0.46**	−0.39**	−0.47**	0.21**	−0.30**
% families below poverty a	−0.53**	−0.45**	−0.55**	0.26**	−0.36**
% below 138% poverty a	−0.54**	−0.45**	−0.56**	0.27**	−0.36**
% single-parent families a	−0.52**	−0.43**	−0.54**	0.28**	−0.34**
% households w/out motor vehicle a	−0.39**	−0.32**	−0.42**	0.20**	−0.23**
% households w/out telephone a	−0.22**	−0.20**	−0.23**	0.10**	−0.13**
% households w/out plumbing a	−0.11**	−0.09**	−0.10**	0.05**	−0.07**
weighted sum (Kind 2014) a	−0.35**	−0.29**	−0.37**	0.19**	−0.27**
national percentile a	−0.50**	−0.39**	−0.52**	0.30**	−0.37**
Population density (UN-adjusted) a	−0.16**	−0.15**	−0.17**	0.06**	−0.05**
Total crime b	−0.03**	−0.09**	−0.03*	0.01	0.06**
Total adult offenses b	−0.02	−0.08**	−0.01	0.00	0.07**
Adult violent crimes b	−0.02*	−0.09**	−0.02*	0.00	0.07**
Drug abuse violations (total) b	−0.05**	−0.12**	−0.05**	0.01	0.05**
Drug sale (total) b	−0.06**	−0.13**	−0.06**	0.02	0.05**
Marijuana sale b	−0.01	−0.08**	−0.01	0.00	0.07**
Drug possession (total) b	−0.05**	−0.12**	−0.04**	0.01	0.06**
Driving under influence DUI b	−0.02	−0.09**	−0.01	−0.01	0.07**
**White**					
% adults < 9 years education a	−0.36**	−0.37**	−0.36**	0.14**	−0.23**
% ≥ high school diploma	0.48**	0.46**	0.48**	−0.21**	0.32**
% white-collar occupations a	0.10**	0.13**	0.07**	−0.02*	0.12**
share of households ≥ $50k a	0.44**	0.37**	0.45**	−0.23**	0.30**
median family income a	0.52**	0.45**	0.54**	−0.26**	0.36**
income disparity (Singh) a	−0.47**	−0.38**	−0.51**	0.25**	−0.29**
median home value a	0.38**	0.30**	0.39**	−0.21**	0.31**
median gross rent a	0.27**	0.21**	0.28**	−0.16**	0.22**
median monthly mortgage a	0.40**	0.32**	0.42**	−0.22**	0.33**
% owner-occupied a	0.36**	0.32**	0.40**	−0.19**	0.19**
% crowding (>1 person/room) a	−0.27**	−0.30**	−0.27**	0.10**	−0.18**
% unemployed a	−0.46**	−0.39**	−0.47**	0.21**	−0.30**
% families below poverty a	−0.53**	−0.45**	−0.55**	0.26**	−0.36**
% below 138% poverty a	−0.54**	−0.45**	−0.56**	0.27**	−0.36**
% single-parent families a	−0.52**	−0.43**	−0.54**	0.28**	−0.34**
% households w/out motor vehicle a	−0.39**	−0.32**	−0.42**	0.20**	−0.23**
% households w/out telephone a	−0.22**	−0.20**	−0.23**	0.10**	−0.13**
% households w/out plumbing a	−0.11**	−0.09**	−0.10**	0.05**	−0.07**
weighted sum (Kind 2014) a	−0.35**	−0.29**	−0.37**	0.19**	−0.27**
national percentile a	−0.50**	−0.39**	−0.52**	0.30**	−0.37**
Population density (UN-adjusted) a	−0.16**	−0.15**	−0.17**	0.06**	−0.05**
Total crime b	−0.03**	−0.09**	−0.03*	0.01	0.06**
Total adult offenses b	−0.02	−0.08**	−0.01	0.00	0.07**
Adult violent crimes b	−0.02*	−0.09**	−0.02*	0.00	0.07**
Drug abuse violations (total) b	−0.05**	−0.12**	−0.05**	0.01	0.05**
Drug sale (total) b	−0.06**	−0.13**	−0.06**	0.02	0.05**
Marijuana sale b	−0.01	−0.08**	−0.01	0.00	0.07**
Drug possession (total) b	−0.05**	−0.12**	−0.04**	0.01	0.06**
Driving under influence DUI b	−0.02	−0.09**	−0.01	−0.01	0.07**
**Black**					
% adults < 9 years education a	−.162**	−.112**	−.163**	.025	−.114**
% ≥ high school diploma a	.352**	.298**	.333**	−.090**	.254**
% white-collar occupations a	−.033	−.046*	−.024	.005	.019
share of households ≥ $50k a	.414**	.331**	.391**	−.147**	.324**
median family income a	.482**	.408**	.474**	−.161**	.351**
income disparity (Singh) a	−.499**	−.416**	−.493**	.157**	−.357**
median home value a	.377**	.308**	.365**	−.183**	.308**
median gross rent a	.389**	.319**	.384**	−.158**	.308**
median monthly mortgage a	.419**	.340**	.413**	−.192**	.337**
% owner-occupied a	.347**	.296**	.348**	−.130**	.231**
% crowding (>1 person/room) a	−.052*	−.002	−.051*	.026	−.043
% unemployed a	−.396**	−.344**	−.375**	.092**	−.297**
% families below poverty a	−.461**	−.386**	−.450**	.126**	−.345**
% below 138% poverty a	−.482**	−.398**	−.471**	.138**	−.360**
% single-parent families a	−.468**	−.404**	−.449**	.168**	−.348**
% households w/out motor vehicle a	−.418**	−.364**	−.404**	.125**	−.286**
% households w/out telephone a	−.157**	−.139**	−.141**	.044*	−.114**
% households w/out plumbing a	−.105**	−.093**	−.091**	.022	−.073**
ADI, weighted sum (Kind 2014) a	−.242**	−.195**	−.230**	.123**	−.174**
ADI, national percentile a	−.420**	−.337**	−.403**	.195**	−.315**
Population density (UN-adjusted) a	−.108**	−.106**	−.102**	.012	−.022
Total crime b	.099**	.056**	.096**	−.030	.173**
Total adult offenses b	.126**	.084**	.121**	−.040	.188**
Adult violent crimes b	.115**	.070**	.114**	−.049*	.181**
Drug abuse violations (total) b	.053*	.002	.057*	−.030	.141**
Drug sale (total) b	.028	−.024	.035	−.022	.128**
Marijuana sale b	.128**	.083**	.122**	−.038	.193**
Drug possession (total) b	.058*	.008	.061**	−.031	.144**
Driving under influence DUI b	.125**	.086**	.121**	−.043*	.180**

**Note.** Values are Pearson correlation coefficients (r), rounded to two decimals.

* p < .05,

** p < .01 (two-tailed).

a Census Track, an Area Deprivation Index (ADI) element;

b Census Track, a Universal Crime Report (UCR) element

**Table 5.** Summary of linear regressions between race/ethnicity, Family SEP, and Neighborhood Variables.

Neighborhood Indicator	High SEP β	Black β	High SEP x Black β

% households with income ≥ $50,000 ^a^	0.228**	−0.329**	0.092**
Median family income ^a^	0.379**	−0.223**	0.001
Income disparity index ^a^	−0.236**	0.387**	−0.121**
Median home value ^a^	0.263**	−0.193**	0.020
Median gross rent ^a^	0.161**	−0.179**	0.079**
Median monthly mortgage ^a^	0.274**	−0.211**	0.053**
% owner-occupied homes ^a^	0.191**	−0.277**	0.066**
% unemployed (age ≥16) ^a^	−0.196**	0.434**	−0.139**
% families below poverty line ^a^	−0.261**	0.402**	−0.134**
% population below 138% poverty threshold ^a^	−0.296**	0.365**	−0.106**
% single-parent households ^a^	−0.214**	0.502**	−0.145**
% households without a vehicle ^a^	−0.089**	0.472**	−0.197**
% households without a telephone ^a^	−0.128**	0.152**	−0.021
% households without complete plumbing ^a^	−0.047**	0.079**	−0.028*
Area Deprivation Index weighted sum ^a^	−0.270**	0.145**	0.019
ADI national percentile (higher = more deprived) ^a^	−0.312**	0.354**	−0.078**
% adults with <9 years of education ^a^	−0.309**	−0.005	0.076**
% adults with ≥ high school diploma ^a^	0.347**	−0.153**	−0.006
% employed in white-collar occupations ^a^	0.178**	0.193**	−0.078**
% crowded housing (>1 person/room) ^a^	−0.285**	−0.089**	0.097**
Population density ^a^	−0.114**	0.050**	0.021
Total crime rate ^a^	−0.038**	−0.019	0.054**
Adult total offenses ^a^	−0.041**	−0.044**	0.064**
Adult violent crimes ^a^	−0.047**	−0.046**	0.063**
Total drug violations ^a^	−0.043**	0.003	0.041**
Drug sales ^a^	−0.044**	0.016	0.032*
Marijuana sales ^a^	−0.029*	−0.029*	0.060**
Drug possession ^a^	−0.043**	0.000	0.043**
DUI incidents ^a^	−0.046**	−0.057**	0.062**

* p < .05

** p < .01 (Standardized coefficients) Models also controlled for age (months) and sex (male).

a (census track, Area Deprivation Index [ADI])
